# Disparities in Pain Evaluation and Treatment During Labor: A Racial and Ethnic Perspective

**DOI:** 10.3390/jcm14093097

**Published:** 2025-04-30

**Authors:** Namrata N. Vasquez, Pedro T. Ramirez, Chandra Bautista, Alok Madan, Jessica C. Rohr

**Affiliations:** 1 Houston Methodist Academic Institute, Houston, TX 77030, USA; 2 Weill Cornell Medical College, New York, NY 10065, USA

**Keywords:** pain, maternal morbidity, childbirth, labor, pain disparities

## Abstract

**Background/Objectives:** In the United States, maternal morbidity is 2–3 times higher than in other high-income nations and continues to rise among non-White women. One potential driving factor is whether labor and childbirth pain are assessed and addressed in a timely and effective manner. Pain during labor and childbirth can be symptomatic of maternal morbidity (e.g., pelvic pain, bleeding, high blood pressure, cardiovascular issues) and/or an independent predictor of adverse postpartum outcomes (e.g., chronic postpartum pain, postpartum depression). **Methods:** Since racial and ethnic disparities in pain reporting and treatment are well documented in other settings—such as chronic pain conditions, pregnancy-related pain, and postpartum care—we hypothesize that similar disparities persist during labor and delivery. In this manuscript, we evaluate differences in pain reporting and provider treatment response (or lack thereof) based on self-reported race and ethnicity during childbirth admission. **Results:** In a large, representative sample of women giving birth at a large hospital system (N = 46,671), we assessed race- and ethnicity-related disparities in pain reporting, evaluation, and treatment. There are racial disparities in the frequency of pain assessments, values of pain ratings, and delivery of pharmacological vs. non-pharmacological treatment. **Conclusions:** A large-scale investigation into racial and ethnic differences in pain assessment, reporting, and treatment during childbirth may help identify mechanisms that mitigate disparities in maternal morbidity and mortality.

## 1. Introduction

Labor and delivery pain is common during childbirth admissions [1]. However, severe levels of pain (7/10 or greater) are associated with adverse health outcomes such as depression and chronic back pain [2,3]. The severity of acute postpartum pain is a strong predictor of both persistent postpartum pain and postpartum depression, independent of the mode of delivery [2]. Severe postpartum back pain increases the risk of long-term disability, yet it often remains inadequately treated [4]. In a hospital study, higher pre-delivery pain was associated with a 30% greater risk of postpartum anxiety and/or depression [5]. Even when controlling for prenatal depressive symptoms, atypical pain trajectories during pregnancy are associated with a greater risk of postpartum depression [6], such that increasing pain later in pregnancy is a predictor of more severe postpartum depression, highlighting the importance of pain in both mental and physical recovery following childbirth.

Among non-pregnant individuals, pain perception and treatment are significantly shaped by social determinants of health, including community context, economic stability, healthcare access, and neighborhood conditions. For example, individuals from underserved populations such as racially marginalized or minoritized backgrounds suffer greater severity of pain, more adverse pain outcomes, and less adequate pain treatment than peers across various pain conditions and treatment settings [7,8]. Socioeconomic disadvantage also contributes to disparities in experience of pain, with disparities increasing over the past 20 years [9].

Studies assessing whether these disparities are present in pregnant populations are limited. Across healthcare system studies, Black women report higher pregnancy pain (>5 on 0–10 scale) and postpartum pain [10,11] and lower levels of management with opioid medications both during delivery and at discharge [12] despite potential indication given the pain level reported. Women of color are less likely than White women to receive an epidural and are also less likely to experience effective pain relief from the medication provided [13]. Similarly, in a large sample of women who underwent a Cesarean section, severe pain (7/10 or greater) was more common among Black and Hispanic women than among women who identified as White or Asian; however, White women received more frequent pain assessments in the first 24 h and received more narcotic medication postpartum [14].

To better understand the scope of pain disparities, this study examined pain reporting and treatment in a large diverse sample in the Southeastern United States. This study aligns with recent calls for more inclusive and equitable research with a focus toward improving pain science and combatting longstanding disparities [15]. This manuscript explores disparities in pain assessment, reporting, and provider treatment responses during childbirth admission, based on self-reported race and ethnicity. Outcome variables included frequency of pain assessments, values of pain ratings, and delivery of pharmacological and non-pharmacological treatment.

## 2. Methods

### 2.1. Design and Setting

This cross-sectional study was conducted at Houston Methodist, a hospital system encompassing a flagship tertiary care hospital and 7 community hospitals serving the greater Houston metro area (population of 7.34 million and the most ethnically diverse large city in the United States [16,17]). Each Houston Methodist hospital has a childbirth center, and 6 hospitals have a Houston Methodist OB/GYN outpatient clinic. The hospitals are located across the greater Houston area including suburbs, capturing the childbirth experiences of individuals across diverse socioeconomic, regional, and cultural backgrounds in one of the most diverse regions of the United States. The Houston Methodist Institutional Review Board approved the study and waived the requirement for obtaining informed consent as it presented no more than minimal risk, minimal PHI was accessed, and no direct patient contact would occur (approved 10 September 2021, reference number MOD00003667). Study procedures met Health Insurance Portability and Accountability Act requirements and 42 CFR Part 2. This report follows the Strengthening the Reporting of Observational Studies in Epidemiology (STROBE) guideline for cross-sectional studies [18].

### 2.2. Participants

This study included patients who delivered at Houston Methodist hospitals between 1 January 2020 and 30 November 2023. All data were ascertained from electronic health records (EHRs) via the COVID-19 Surveillance and Outcomes Registry (CURATOR), a research registry. Detailed methods of the CURATOR project have been reported elsewhere [19,20]. Inclusion criteria were as follows: (1) over the age of 18 and (2) had complete data available for all variables of interest. All patients with complete data for variables of interest were included in the data analysis.

### 2.3. Variables and Data Sources

*Demographic information*. Age, biological sex (as listed on driver’s license), insurance type, and patient-reported race and ethnicity were extracted from the registry. Patient-reported race is recorded in the EHR as Caucasian, Black, Asian, Native American, Hawaiian/Pacific Islander, Multiracial, Other, or Decline. Patient-reported ethnicity is reported in EHR as Hispanic or Latino, Not Hispanic or Latino, or Decline. Race and ethnicity were combined into a single variable Black, Hispanic White, non-Hispanic White, Asian, Native American, Hawaiian/Pacific Islander, Multiracial, and Other.

#### Pain Variables

Numerical Rating Scale for Pain. Pain was assessed and recorded by nurses throughout inpatient admission. Pain ratings were reported on a scale of 0–10, with 0 reflecting no pain and 10 reflecting the worst pain imaginable.

Number of assessments. The number of assessments was measured as the number of times a patient’s pain rating was recorded during their inpatient admission for childbirth. We also created a variable for the average number of assessments per day during admission to account for variations in length of stay.

Pain treatment variables. The delivered pain intervention was recorded as part of the nursing pain assessment during the admission for childbirth. There are numerous options for pain intervention that were collapsed into 4 categories: declined intervention, medications, MD notified, no intervention, and comfort measures (acupressure, acupuncture, ambulation/increased activity, aromatherapy, back rub, cold application, cold pack, cutaneous stimulation, distraction, elevation, emotional support, environmental changes, food, guided imagery, heat application, herbal therapy, massage, music, pet therapy, prayers, relaxation technique, repositioning, rest, shower, sitz bath, spiritual care consult, splinting, Transcutaneous Electrical Nerve Stimulation (TENS), therapeutic presence, therapeutic touch, traction, tub bath, warm moist pack, and warm pack). Results for cases labeled as ‘no intervention recorded’ were excluded due to variability in provider documentation and insufficient detail on whether pain treatment was offered and, if so, what type.

Pregnancy variables. The first birth per individual during the study time period was used for analyses. Also extracted from the registry were variables often associated with pain including length of stay and type of delivery.

### 2.4. Data Analysis

Categorical variables were reported as percentages, and continuous variables were reported as means and SDs. Pearson product-moment correlations and one-way ANOVA were used to examine relationships between potential covariates and study variables. Significant results were used to identify covariates. We fit univariable and multivariable regression models to the data to predict pain management from race/ethnic variables, correcting for covariates. Race and ethnicity differences in pain treatment were assessed via univariate general linear models. Analysis of the data was conducted 1 July 2024 through 14 November 2024, using SPSS, version 29.0.2.0 [21].

## 3. Results

### 3.1. Descriptive and Inferential Statistics

**Figure 1:**
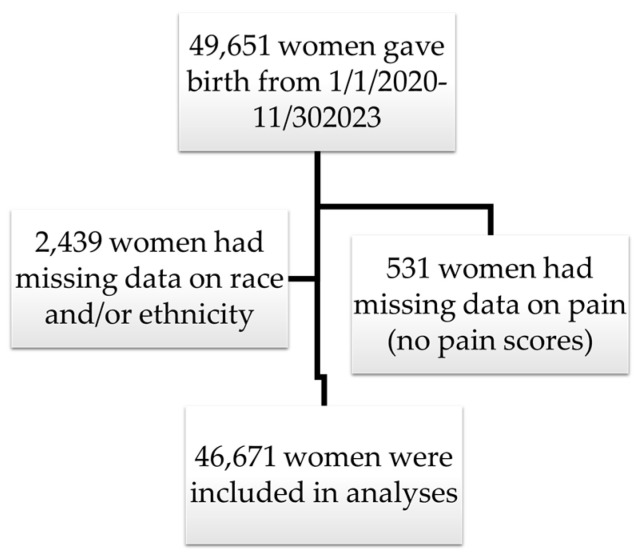
Flow chart of inclusion and exclusion of data.

During the study period, 49,641 women gave birth at a Houston Methodist hospital. A total of 2439 women were removed due to missing data on race or ethnicity. Additionally, 531 additional women with no pain scores were removed. A final 46,671 were eligible for inclusion in our analyses (Figure 1). The number of pain scores recorded per admission ranged from 1 to 1745. Only the first 100 pain scores for each patient were used in analyses because 99% of women had fewer than 100 pain scores recorded.

The mean age of women included in our analyses was 30.04 years (SD = 5.50). See Table 1 for the racial and ethnic breakdown of the sample. The average pain score throughout the inpatient stay was 1.77 (SD = 1.20). The average number of pain assessments during an admission was 28.44 (SD 17.17) and the median number of pain assessments was 26. The average number of pain assessments per day during admission was 11.00 (SD = 5.79). See Table 2 for the relationship between variables often associated with pain and pain scores (continuous) and race/ethnicity (categorical). Age was significantly negatively correlated with average pain scores (*r* = −0.010, *p* = 0.04), such that greater age was associated with lower reported pain. Age also differed significantly between racial and ethnic groups (F = 6.78, *p* < 0.001) and was included in subsequent adjusted models as a covariate.

### 3.2. Univariate General Linear Model (GLM) for Relationship Between Race/Ethnicity and Pain Scores

The univariate general linear model (Table 3) demonstrated that pain scores were significantly different based on race and ethnicity even after correcting for age, length of stay, and whether the patient had a Cesarean delivery (F = 8.11, *p* < 0.001). Post hoc contrasts suggested that Black women had significantly higher pain scores than White women (1.83 vs. 1.77, *p* < 0.001), and Asian and Multiracial women reported significantly lower pain scores than White women (1.68 and 1.63, respectively, both at *p* < 0.001; see Table 3 for all contrast results).

### 3.3. Differences in Pain Treatment

Pain medication. The univariate general linear model (Table 4) demonstrated that reported administration of pain medication differed significantly based on race and ethnicity even after correcting for age, length of stay, whether the patient had a Cesarean delivery, and average pain score (F = 59.43, *p* < 0.001). Post hoc contrasts suggested that Black women, Hispanic White women, Multiracial women, and women reporting their ethnicity as “Other” had a significantly lower likelihood of receiving pain medication than non-Hispanic White women (Table 4).

Comfort measures. The univariate general linear model (Table 5) demonstrated that the reported administration of comfort measures was significantly different based on race and ethnicity even after correcting for age, length of stay, whether the patient had a Cesarean Delivery, and average pain score (F = 27.26, *p* < 0.001). Post hoc contrasts suggested that Asian women had a significantly higher likelihood of comfort measure interventions than non-Hispanic White women (Table 5), while Hispanic White women, Multiracial women, and women identifying as “Other” in terms of race or ethnicity were less likely than White women to receive comfort measures.

Notifying MD of pain. The univariate general linear model (Table 6) demonstrated that notifying an MD provider of pain was not significantly different based on race and ethnicity.

Declining further intervention. The univariate general linear model (Table 7) demonstrated that declining pain intervention was significantly different based on race and ethnicity even after correcting for age, length of stay, whether the patient had a Cesarean Delivery, and average pain scores (F = 58.41, *p* < 0.001). Post hoc contrasts suggested that Black women, Hispanic White women, Asian women, and Native American women were more likely to decline an intervention than White women (Table 7). Both Multiracial women and women identifying as ‘Other’ in terms of race were less likely than White women to decline an intervention.

## 4. Discussion

In a large, representative sample of women admitted for childbirth, significant differences were evident in reported pain, pain evaluation, and pain treatment across racial and ethnic groups. Black women reported significantly more pain than non-Hispanic White women, and Multiracial and Asian women reported significantly less pain than non-Hispanic White women, though overall pain scores were low and may not be clinically significant. Black women were less likely to be prescribed pain medication and more likely to decline pain-related intervention after reporting pain than non-Hispanic White women. Hispanic White women were also less likely than non-Hispanic White women to be prescribed pain medication and to receive comfort measures while more likely to decline pain-related intervention after reporting pain. Asian women had higher rates of receiving comfort measures as well as declining interventions when offered than non-Hispanic White women. Multiracial women and women identifying as Other were less likely to be prescribed pain medication, use comfort measures, and decline intervention compared to non-Hispanic White women.

The racial disparities in pain treatment observed in this study are consistent with previous research on systemic biases across the healthcare system [22], as well as cultural factors involved in pain and childbirth [23] and varying perceptions of medical care across racial and ethnic groups [24]. For example, Asian women may be less likely to report pain in childbirth and prefer less medical intervention due to an array of cultural beliefs, traditions, and taboos around childbirth [25]. In contrast, the experiences of Black and Hispanic White women may be shaped by poor patient–provider relationships in the context of well-documented, systemic patterns of maltreatment such as being ignored or refused when seeking help for pain [26]. Notably, the findings for women identifying as Multiracial and ‘Other’ were less consistent across the variables examined in the study, which may be due to greater heterogeneity compared to the ‘Other’ groups. Additional research is needed to further explore the unique experiences of individuals in these groups.

The current findings demonstrate an urgent need for greater understanding of the psychosocial aspects of patient–provider interactions to ensure patients are receiving needed treatment during childbirth. Appropriate and equitable pain management in the peripartum period would likely help prevent postpartum depression [2] and postpartum chronic pain [27]. Specific educational guidelines and practice policies are needed to promote positive patient–provider relationships and to improve understanding of the cultural context of childbirth pain at the levels of providers, healthcare systems, and communities [28].

Attempts to implement implicit bias training for medical providers have yielded limited effectiveness [29]. Systemic approaches to improving patient–provider relationships and health equity include (1) reforms to medical training that include cultural considerations in medical practice [30], (2) guideline-based assessment and treatment of pain as opposed to standardized protocols that can exacerbate disparities [31], and (3) multimodal, collaborative interventions that target both psychological and pain symptoms in the peripartum period [32]. At the practice level, providers may consider implementing comprehensive standardized pain measures at regular intervals in primary, psychological, and/or peripartum care. Brief psychoeducational interventions focused on pain management, mindfulness, and stress reduction are likely to empower patients to cope with peripartum pain. Additionally, community-based programs—such as those involving doulas and midwives with direct knowledge of relevant cultural factors—alongside culturally competent healthcare providers may be beneficial. However, evidence supporting their effectiveness remains limited [33].

While this study makes important contributions, its limitations should also be acknowledged. First, though the differences in reported pain were statistically significant across racial and ethnic groups, the differences were small and may not be clinically significant. Additionally, the overall reported pain levels were lower than expected, with an overall average of 1.77 on a 0–10 scale. It is important to note that pain was assessed multiple times across the entire hospitalization, including early phases of childbirth and during recovery. However, differences in other variables (e.g., pain treatment) warrant concern. Furthermore, the role of cultural factors and psychosocial attributes of different racial and ethnic identities was not assessed in this study and likely plays a role. The included racial and ethnic categories are also not inclusive of all patient backgrounds and do not capture several identities that may have better aligned with patient backgrounds. Second, the pain scale used by clinicians may not reflect the full range of labor pain experiences and is limited in nature. Pain scores may also not have been recorded appropriately to be extracted during the chart review in this study. Third, the sample included childbirths that occurred during the COVID-19 pandemic, which may have played a role in maternal morbidity [34], though additional exploration of this is needed. The sample was not adjusted for pre- versus post-pandemic births, but we hope to explore this variable in future analyses. Fourth, several relevant variables such as prior chronic pain conditions and pregnancy complications were beyond the scope of the current manuscript but warrant further analysis and discussion. There is variability in epidural indications, and variable use across providers may confound results. We also do not have details on the anesthetics used. We have described the different methods of anesthesia used in C-sections, and a more in-depth examination of anesthetic choice is warranted in future studies. Furthermore, the pain medication administered may also vary across provider, and medication specificity was not available for analysis. Finally, as the data were extracted from medical charts, they could not be clarified or verified—such as determining the specific racial and ethnic composition of the ‘Multiracial’ and ‘Other’ groups—thereby limiting the specificity of some findings.

In summary, the present study highlights significant racial and ethnic disparities in pain evaluation and treatment during childbirth. These findings underscore the need for systemic changes in healthcare practices to ensure equitable care. Future research should explore the cultural, social, and institutional factors driving these disparities, with an emphasis on developing targeted interventions that enhance provider education and integrate culturally competent care. By fostering more inclusive and responsive healthcare systems, we can ensure that all women receive appropriate and compassionate pain management during childbirth and lower the risk of subsequent chronic pain conditions.

## Figures and Tables

**Table 1:** Average pain rating at first admission for delivery for each race or ethnicity group.

Race or Ethnicity Identification (Self-Report)	N	Mean Pain Rating	Std. Deviation of Pain Rating
Asian	5002	1.6783	1.12186
Black	8358	1.8322	1.29719
Hispanic White	13,557	1.7694	1.19172
Non-Hispanic White	17,873	1.7663	1.17354
Hawaiian-Pacific Islander	136	1.8049	1.39855
Multiracial	1182	1.6312	1.25976
Native American	462	1.8681	1.29773
Other	108	1.6372	1.51495
Total	46,678	1.7670	1.20241

Note. Pain rating ranges from 0–10.

**Table 2:** Relationship between variables of interest and covariates.

	Average Pain Score During Admission ^1^		Race/Ethnicity ^2^	
	*r*	*p*	F	*p*
Age at delivery	−0.010	0.039	297.43	<0.001 *
Length of stay (in hours)	0.014	0.003	17.37	<0.001 *
Cesarean Delivery	0.105	<0.001 *	15.03	<0.001 *

Note. * Significant at *p* < 0.001. ^1^ Pearson product-moment correlation was used to examine relationships between continuous pain score variable and covariates. ^2^ One-way ANOVA was used to examine relationships between categorical race/ethnicity variable and covariates.

**Table 3:** Corrected general linear model predicting pain scores from race/ethnicity.

**Factor**	**Type III SS**	**F**	***p*-Value**
Corrected model	875.83	61.36	<0.001 *
Intercept	3135.31	2196.65	<0.001 *
Age	33.27	23.31	<0.001 *
LOS	9.10	6.37	0.012
Cesarean Delivery	760.28	532.66	<0.001 *
Race/Ethnicity	81.04	8.11	<0.001 *
**Contrasts (M, SD)**	**Contrast Estimate**	**CI**	***p*-Value**
Non-Hispanic White (1.77, 1.17)	Reference group	n/a	n/a
Black (1.83, 1.29)	0.052	0.020–0.083	<0.001 *
Hispanic White (1.77, 1.19)	0.000	−0.026–0.027	0.976
Asian (1.68, 1.12)	−0.081	−0.118–0.043	<0.001 *
Hawaiian-Pacific Islander (1.80, 1.40)	0.061	−0.141–−0.262	0.555
Multiracial (1.63, 1.26)	−0.137	−0.207–−0.067	<0.001 *
Native American (1.87, 1.30)	0.083	−0.027–0.194	0.140
Other (1.64, 1.51)	−0.143	−0.369–0.083	0.214

Note. * *p* < 0.001. The top half of the table displays results of the univariate GLM predicting pain scores from race/ethnicity including age, length of stay, and Cesarean delivery as covariates. The bottom half of the table displays results of post hoc comparisons between race/ethnicity categories using non-Hispanic White as the reference group. Pain scores for all other race/ethnicity categories are compared to those for non-Hispanic White. The contrast estimate, confidence interval (CI), and *p*-value (significance of the contrast) are presented.

**Table 4:** Univariate GLM for relationship between race/ethnicity and pain medication.

**Factor**	**Type III SS**	**F**	***p*-Value**
Corrected model	85.086	634.40	<0.001 *
Intercept	5.013	411.12	<0.001 *
Age	0.741	60.79	<0.001 *
LOS	1.151	94.43	<0.001 *
Cesarean Delivery	20.936	1717.09	<0.001 *
Average pain score	51.303	4207.67	<0.001 *
Race/Ethnicity	5.68	66.57	<0.001 *
**Contrasts (M, SD)**	**Contrast Estimate**	**CI**	***p*-Value**
Non-Hispanic White (0.130, 1.22)	Reference group	n/a	n/a
Black (0.111, 0.118)	−0.023	(−0.025, −0.020)	<0.001 *
Hispanic White (0.121, 0.115)	−0.009	(−0.012, −0.007)	<0.001 *
Asian (0.130, 0.115)	0.004	(0.001−0.008)	0.018
Hawaiian-Pacific Islander (0.117, 0.129)	−0.009	(−0.028, 0.009)	0.321
Multiracial (0.085, 0.111)	−0.040	(−0.047, −0.034)	<0.001 *
Native American (0.140, 0.120)	0.004	(−0.006, 0.015)	0.397
Other (0.037, 0.080)	−0.092	(−0.113, −0.071)	<0.001 *

Note. * *p* < 0.001. The top half of the table displays results of the univariate GLM predicting pain medication from race/ethnicity including age, length of stay, Cesarean delivery, and average pain score as covariates. The bottom half of the table displays results of post hoc comparisons between race/ethnicity categories using non-Hispanic White as the reference group. Pain scores for all other race/ethnicity categories are compared to those for non-Hispanic White. The contrast estimate, confidence interval (CI), and *p*-value (significance of the contrast) are presented.

**Table 5:** Univariate GLM for relationship between race/ethnicity and delivery of comfort measures.

**Factor**	**Type III SS**	**F**	***p*-Value**
Corrected model	9.319	161.13	<0.001 **
Intercept	0.699	133.01	<0.001 **
Age	0.087	16.50	<0.001 **
LOS	0.034	6.43	0.011
Cesarean Delivery	0.33	62.58	<0.001 **
Average pain score	7.61	1447.57	<0.001 **
Race/Ethnicity	1.003	27.26	<0.001 **
**Contrasts (M, SD)**	**Contrast Estimate**	**CI**	***p*-Value**
Non-Hispanic White (0.045, 0.073)	Reference group	n/a	n/a
Black (0.044, 0.076)	−0.002	(−0.004, 0.000)	0.083
Hispanic White (0.043, 0.072)	−0.002	(−0.004, −0.001)	0.005 *
Asian (0.054, 0.079)	0.011	(0.009, 0.013)	<0.001 **
Hawaiian-Pacific Islander (0.035, 0.065)	−0.010	(−0.022, 0.003)	0.125
Multiracial (0.033, 0.067)	−0.010	(−0.015, −0.006)	<0.001 **
Native American (0.055, 0.082)	0.008	(0.001, 0.015)	0.017
Other (0.006, 0.026)	−0.037	(−0.051, −0.023)	<0.001 **

Note. * *p* < 0.01; ** *p* < 0.001. The top half of the table displays results of the univariate GLM predicting comfort measures from race/ethnicity including age, length of stay, Cesarean delivery, and average pain score as covariates. The bottom half of the table displays results of post hoc comparisons between race/ethnicity categories using non-Hispanic White as the reference group. Pain scores for all other race/ethnicity categories are compared to those for non-Hispanic White. The contrast estimate, confidence interval (CI), and *p*-value (significance of the contrast) are presented.

**Table 6:** Univariate GLM for relationship between race/ethnicity and notification to MD provider.

Factor	Type III SS	F	*p*-Value
Corrected model	0.008	20.13	<0.001 **
Intercept	0.001	22.08	<0.001 **
Age	0.001	16.75	<0.001 **
LOS	0.000	8.01	0.005 *
Cesarean Delivery	0.003	77.43	<0.001 **
Average pain score	0.004	111.70	<0.001 **
Race/Ethnicity	0.000	1.54	0.148

Note. * *p* < 0.01; ** *p* < 0.001. The top half of the table displays results of univariate the GLM predicting notification of MD from race/ethnicity including age, length of stay, Cesarean delivery, and average pain score as covariates. No post hoc contrasts were conducted as the prediction of notification of an MD from race/ethnicity was not significant.

**Table 7:** Univariate GLM for relationship between race/ethnicity and declining intervention.

**Factor**	**Type III SS**	**F**	***p*-Value**
Corrected model	8.73	91.59	<0.001 **
Intercept	3.09	356.89	<0.001 **
Age	0.16	18.51	<0.001 **
LOS	0.48	55.55	<0.001 **
Cesarean Delivery	0.15	17.26	<0.001 **
Average pain score	4.54	523.81	<0.001 **
Race/Ethnicity	3.54	58.41	<0.001 **
**Contrasts (M, SD)**	**Contrast Estimate**	**CI**	***p*-Value**
Non-Hispanic White (0.056, 0.087)	Reference group	n/a	n/a
Black (0.060, 0.096)	0.004	(0.001, 0.006)	<0.001 **
Hispanic White (0.061, 0.095)	0.004	(0.002, 0.006)	<0.001 **
Asian (0.082, 0.108)	0.027	(0.024, 0.030)	<0.001 **
Hawaiian-Pacific Islander (0.059, 0.093)	0.003	(−0.013, 0.019)	0.715
Multiracial (0.043, 0.080)	−0.012	(−0.018, −0.007)	<0.001 **
Native American (0.070, 0.102)	0.013	(0.004, 0.022)	0.003 *
Other (0.010, 0.031)	−0.045	(−0.062, −0.027)	<0.001 **

Note. * *p* < 0.01; ** *p* < 0.001. The top half of the table displays results of the univariate GLM predicting declining pain intervention from race/ethnicity including age, length of stay, Cesarean delivery, and average pain score as covariates. The bottom half of the table displays results of post hoc comparisons between race/ethnicity categories using non-Hispanic White as the reference group. Pain scores for all other race/ethnicity categories are compared to those for non-Hispanic White. The contrast estimate, confidence interval (CI), and *p*-value (significance of the contrast) are presented.

## Data Availability

The data presented in this study are not readily available to protect healthcare information and integrity.

## References

[1] Lowe N.K. (2002). The nature of labor pain. Am. J. Obstet. Gynecol..

[2] Eisenach J.C., Pan P.H., Smiley R., Lavand’homme P., Landau R., Houle T.T. (2008). Severity of acute pain after childbirth, but not type of delivery, predicts persistent pain and postpartum depression. Pain.

[3] Zhang J., Troendle J., Mikolajczyk R., Sundaram R., Beaver J., Fraser W. (2010). The natural history of the normal first stage of labor. Obstet. Gynecol..

[4] Zhang M., Cooley C., Ziadni M.S., Mackey I., Flood P. (2023). Association between history of childbirth and chronic, functionally significant back pain in later life. BMC Women’s Health.

[5] Tan H.S., Agarthesh T., Tan C.W., Sultana R., Chen H.Y., Chua T.E., Sng B.L. (2021). Perceived stress during labor and its association with depressive symptomatology, anxiety, and pain catastrophizing. Sci. Rep..

[6] Mathur V.A., Nyman T., Nanavaty N., George N., Brooker R.J. (2021). Trajectories of pain during pregnancy predict symptoms of postpartum depression. PAIN Rep..

[7] Hobson J.M., Moody M.D., Sorge R.E., Goodin B.R. (2022). The neurobiology of social stress resulting from Racism: Implications for pain disparities among racialized minorities. Neurobiol. Pain.

[8] Mathur V.A., Trost Z., Ezenwa M.O., Sturgeon J.A., Hood A.M. (2022). Mechanisms of injustice: What we (do not) know about racialized disparities in pain. Pain.

[9] Zajacova A., Grol-Prokopczyk H., Zimmer Z. (2021). Sociology of Chronic Pain. J. Health Soc. Behav..

[10] Badreldin N., Ditosto J.D., Grobman W.A., Yee L.M. (2023). Maternal psychosocial factors associated with postpartum pain. Am. J. Obstet. Gynecol. MFM.

[11] Thomas D.A., Nahin R.L. (2022). Cross-Sectional Analyses of High-Impact Pain Across Pregnancy Status by Race and Ethnicity. J. Women’s Health.

[12] Badreldin N., Grobman W.A., Yee L.M. (2019). Racial Disparities in Postpartum Pain Management. Obstet. Gynecol..

[13] Morris T., Schulman M. (2014). Race inequality in epidural use and regional anesthesia failure in labor and birth: An examination of women’s experience. Sex. Reprod. Healthc..

[14] Johnson J.D., Asiodu I.V., McKenzie C.P., Tucker C., Tully K.P., Bryant K., Verbiest S., Stuebe A.M. (2019). Racial and Ethnic Inequities in Postpartum Pain Evaluation and Management. Obstet. Gynecol..

[15] Booker S.Q., Bartley E.J., Powell-Roach K., Palit S., Morais C., Thompson O.J., Cruz-Almeida Y., Fillingim R.B. (2021). The Imperative for Racial Equality in Pain Science: A Way Forward. J. Pain.

[16] Frey W. (2022). A 2020 Census Portrait of America’s Largest Metro Areas: Population growth, diversity, segregation, and youth. Policy Briefs and Reports. https://digitalscholarship.unlv.edu/brookings_policybriefs_reports/11.

[17] Strait J.B., Gong G. (2010). Ethnic Diversity in Houston, Texas: The Evolution of Residential Segregation in the Bayou City, 1990–2000. Popul. Rev..

[18] Ghaferi A.A., Schwartz T.A., Pawlik T.M. (2021). STROBE Reporting Guidelines for Observational Studies. JAMA Surg..

[19] Hagan K., Javed Z., Cainzos-Achirica M., Elizondo J.V., Nicolas C., Yahya T., Acquah I., Blankstein R., Hyder A., Mossialos E. (2021). Abstract 11803: Area Deprivation and COVID-19 Outcomes in Patients With and Without Cardiovascular Disease: The Curator Registry of Houston Methodist. Circulation.

[20] Vahidy F., Jones S.L., Tano M.E., Nicolas J.C., Khan O.A., Meeks J.R., Pan A.P., Menser T., Sasangohar F., Naufal G. (2021). Rapid Response to Drive COVID-19 Research in a Learning Health Care System: Rationale and Design of the Houston Methodist COVID-19 Surveillance and Outcomes Registry (CURATOR). JMIR Med. Inform..

[21] IBM Corp. (2023). IBM SPSS Statistics for Windows, Version 29.0.2.0.

[22] Vedam S., Stoll K., Taiwo T.K., Rubashkin N., Cheyney M., Strauss N., McLemore M., Cadena M., Nethery E., Rushton E. (2019). The Giving Voice to Mothers study: Inequity and mistreatment during pregnancy and childbirth in the United States. Reprod. Health.

[23] Callister C., Khalaf I., Semenic S., Kartchner R., Vehvilainen-Julkunen K. (2003). The pain of childbirth: Perceptions of culturally diverse women. Pain Manag. Nurs..

[24] Saluja B., Bryant Z. (2021). How Implicit Bias Contributes to Racial Disparities in Maternal Morbidity and Mortality in the United States. J. Women’s Health.

[25] Withers M., Kharazmi N., Lim E. (2018). Traditional beliefs and practices in pregnancy, childbirth and postpartum: A review of the evidence from Asian countries. Midwifery.

[26] Komatsu R., Ando K., Flood P.D. (2020). Factors associated with persistent pain after childbirth: A narrative review. Br. J. Anaesth..

[27] Hampton S.B. (2017). The Influence of Race and Gender on Nursing Pain Management Decisions.

[28] Njoku A., Evans M., Nimo-Sefah L., Bailey J. (2023). Listen to the Whispers before They Become Screams: Addressing Black Maternal Morbidity and Mortality in the United States. Healthcare.

[29] Payne B.K., Vuletich H.A., Lundberg K.B. (2017). The bias of crowds: How implicit bias bridges personal and systemic prejudice. Psychol. Inq..

[30] Green T.L., Zapata J.Y., Brown H.W., Hagiwara N. (2021). Rethinking Bias to Achieve Maternal Health Equity: Changing Organizations, Not Just Individuals. Obs. Gynecol..

[31] Cronin K.A., Howlader N., Stevens J.L., Trimble E.L., Harlan L.C., Warren J.L. (2019). Racial disparities in the receipt of guideline care and cancer deaths for women with ovarian cancer. Cancer Epidemiol. Biomark. Prev..

[32] Ray-Griffith S.L., Wendel M.P., Stowe Z.N., Magann E.F. (2018). Chronic pain during pregnancy: A review of the literature. Int. J. Women’s Health.

[33] Kathawa C.A., Arora K.S., Zielinski R., Low L.K. (2022). Perspectives of doulas of color on their role in alleviating racial disparities in birth outcomes: A qualitative study. J. Midwifery Women’s Health.

[34] Westgren M., Pettersson K., Hagberg H., Acharya G. (2020). Severe maternal morbidity and mortality associated with COVID-19: The risk should not be downplayed. Acta Obstet. Gynecol. Scand..

